# Women using hormonal contraceptives show increased valence ratings and memory performance for emotional information

**DOI:** 10.1038/s41386-019-0362-3

**Published:** 2019-03-05

**Authors:** Klara Spalek, Eva Loos, Nathalie Schicktanz, Francina Hartmann, Dominique de Quervain, Christina Stier, Annette Milnik

**Affiliations:** 1 Division of Cognitive Neuroscience, Department of Psychology, University of Basel, Basel, Switzerland; 2 Division of Molecular Neuroscience, Department of Psychology, University of Basel, Basel, Switzerland; 3 Psychiatric University Clinics, University of Basel, Basel, Switzerland; 4 Transfaculty Research Platform, University of Basel, Basel, Switzerland; 5 Clinic of Clinical Neurophysiology, Georg-August University Göttingen, Göttingen, Germany; 6 Department of Neurology and Epileptology, Hertie Institute of Clinical Brain Research, University of Tübingen, Tübingen, Germany

**Keywords:** Perception, Human behaviour

## Abstract

Perception of emotional valence and emotional memory performance vary across the menstrual cycle. However, the consequences of altered ovarian hormone levels due to the intake of hormonal contraceptives on these emotional and cognitive processes remain to be established. In the present study, which included 2169 healthy young females, we show that hormonal contraceptives (HC) users rated emotional pictures as more emotional than HC-non-users and outperformed non-users in terms of better memory recall of emotional pictures. The observed association between HC-status and memory performance was partially mediated by the perception of emotional picture valence, indicating that increased valence ratings of emotional pictures in HC-users led to their better emotional memory performance. These findings extend the knowledge about the relation of HC-intake with emotional valence perception and emotional memory performance. Further, the findings might stimulate further research investigating the interrelation of enhanced memory for emotional events and the increased risk for anxiety-related psychiatric disorders in women.

## Introduction

Puberty, pregnancy, postpartum, and menopause constitute prominent phases in a woman’s life and are accompanied by significant variations in ovarian hormone levels [1]. These major hormonal transition periods seem to be critical time points for the increase in prevalence rates of mood disorders like major depression [1, 2]. Additionally, by comparison to men, women are more likely to develop mood and anxiety disorders [3–6]. In patients with these psychiatric disorders the dysregulation of emotional processes is one of the core symptoms giving rise to the respective diagnosis [7, 8]. Further, a dysregulation of emotional memory capacity constitutes a risk factor for anxiety disorders [9]. In general though, an enhanced memory for emotional events has an obvious adaptive value in evolutionary terms, as it is vital to remember both dangerous and favourable situations [10, 11]. Multiple factors contribute to this well-known emotional memory enhancement effect, one of which is sex [12].

Given the sex-specific risk increase for mood disorders, an increasing body of research focuses on the investigation of ovarian hormone effects on a wide range of emotional processes and emotional memory functions. The menstrual cycle with its natural hormonal variations allows investigating the effects of fluctuations in ovarian hormone levels on various emotional and cognitive processes [13–15]. Overall, the results from those studies show increased memory recall performance for emotional items [13, 14], decreased emotion recognition accuracy [14, 15], a tendency for more intense perception of fearful expressions (adverted vs. direct gaze) [14]‚ and faster reaction to negative stimuli [14] in phases of high hormone (both progesterone and estradiol) levels.

Given those effects the question arises if a pharmacological manipulation of ovarian hormone levels, e.g. through the intake of hormonal contraceptives (HC) also has an influence on emotional processes and emotional memory functions. HC, mostly oral contraceptives (OC), are used worldwide mainly to prevent pregnancy by lowering endogenous ovarian hormone levels and suppressing their fluctuations [16]. For instance in Switzerland every fourth woman between 15 and 49 years of age uses OC [17]. Even though the use of OC is widespread, there is little and inconsistent knowledge about their influence on emotional and cognitive processes [15, 18]. In a recent review, Montoya and Bos [15] highlight the impact of OC on social–emotional behaviours, and report impaired fear extinction, altered emotional memory, and reduced emotion recognition accuracy in OC-users. Specifically, when it comes to differences in emotional memory, women using HC show enhanced memory for gist information but not details of an emotional story compared to a neutral story, whereas in contrast HC-non-users remember more details than gist information [19, 20]. In another study, recall performance of emotional (positive and negative) images differed between women using HC and HC-non-users depending on their stress hormone (noradrenergic and cortisol) response [21]. Further, Hamstra et al. [22] report better memory recall of words describing negative personality characteristics in HC-users compared to HC-non-users. The results from Petersen and Cahill [23] suggest that OC modulation of amygdala reactivity may exert an influence on amygdala-dependent behaviours like emotional memory.

To summarise, several studies find that ovarian hormone variations throughout the menstrual cycle alter emotional memory and processing [13–15]. Few studies show that the intake of HC influences emotional memory [15, 19, 21, 22]. Concerning emotional memory, results point to increased memory for emotional information in menstrual cycle phases with high hormone levels and in HC-users. Overall, these results suggest that an increase in ovarian hormone levels during menstrual cycle, as well as the intake of these hormones through HC might increase the memory boost for emotional information. Regarding emotional processing, results are inconsistent [14, 24–26]. Importantly, it might be possible that the increase in emotional memory performance is due to differences when processing the emotional information. To our knowledge, we are the first ones to investigate differences between HC-users and non-users in the perception of emotional valence and subsequent episodic memory performance, as well as the relationship between differences in these two processes, based on data from a large group (*N* = 2169) of healthy young women.

## Materials and methods

### Participants

We analysed data of *N* = 2169 subjects from two different samples (for an overview of sample characteristics see Table 1). Overall, at the time point of the study 56% of the subjects used hormonal contraceptives (HC-yes) and the rest were naturally cycling women (HC-no). Group allocation to HC-yes or HC-no was based on self-reported information. For the HC-no group information about cycle phase was missing in both samples. Information about the type of hormonal contraception was only available for sample 1. In this sample, out of the 520 women using HC, 430 used OC (83%) and 89 used other methods like ring (*n* = 60, 11%), spiral (*n* = 14, 3%), patch (*n* = 9, 2%), shot (*n* = 3, 0.6%), or rod tabfig(*n* = 3, 0.6%). Information on the specific compounds used was missing in both samples. Across both samples, the mean age was 22.77 years (range 18–35 years). The HC-groups differed significantly in age (*t*_(2167)_ = 3.09, *p* = 0.002) and two scales of the NEO-Five Factor Inventory [27] (NEO-Conscientiousness: *t*_(1648)_ = −5.29, *p* = 1.4 × 10^*−*07^; NEO-Openness to Experience: *t*_(1648)_ = 5.3, *p* = 1.3 × 10^−07^). Meaning that HC-users were younger, more conscientious, and less open to experience. NEO-FFI data were available for a subset of subjects (*n* = 1650) of the overall sample. Subjects were recruited from the area of Basel in Switzerland. Sampling strategy was to recruit large samples of healthy young adults, without further restrictions. Advertising was done mainly at the University of Basel and in local newspapers. Subjects were free of any neurological or psychiatric illness, and did not take any medication (apart from oral contraceptives) at the time of the experiment. The ethics committee of the Canton Basel approved the experiments. Written informed consent was obtained from all subjects before participation.

**Table 1:** Sample characteristics

	Sample 1	Sample 2	All
*N*_total_	902	1267	2169
HC-yes (%)	58	53	56
*Age*			
Range	18–35	18–35	18–35
Mean age ± SD (over both groups)	22.70 ± 3.21	22.82 ± 3.52	22.77 ± 3.39
Mean age ± SD (HC-yes/HC-no)	22.60 ± 2.89/22.83 ± 3.60 *t*_(900)_ = 1.06, *p* = 0.291	22.54 ± 3.12/23.14 ± 3.89 *t*_(1265)_ = 3.03, *p* = 0.002	22.57 ± 3.02/23.02 ± 3.78 *t*_(2167)_ = 3.09, *p* = 0.002
*NEO-FFI scales mean* *±* *SD (HC-yes/HC-no)*			
n HC-yes/n HC-no	245/188	649/568	894/756
Agreeableness (NEO-A)	32.17 ± 5.45/32.96 ± 5.44 *t*_(431)_ = 1.50, *p* = 0.135	32.76 ± 5.43/32.85 ± 5.15 *t*_(1215)_ = 0.27, *p* = 0.787	32.60 ± 5.44/32.87 ± 5.22 *t*_(1648)_ = 1.04, *p* = 0.300
Conscientiousness (NEO-C)	32.75 ± 6.82/31.35 ± 7.15 *t*_(431)_ = −2.08, *p* = 0.039	32.19 ± 6.67/30.29 ± 6.96 *t*_(1215)_ = −4.86, *p* = 1.3 × 10^−06^	32.34 ± 6.71/30.55 ± 7.02 *t*_(1648)_ = −5.29, *p* = 1.4 × 10^−07^
Extraversion (NEO-E)	29.79 ± 5.81/29.8 ± 6.90 *t*_(431)_ = 0.02, *p* = 0.985	30.5 ± 5.96/29.68 ± 6.09 *t*_(1215)_ = −2.36, *p* = 0.018	30.31 ± 5.92/29.71 ± 6.3 *t*_(1648)_ = −1.97, *p* = 0.049
Neuroticism (NEO-N)	22.11 ± 7.14/21.27 ± 7.26 *t*_(431)_ = −1.22, *p* = 0.225	20.54 ± 7.33/20.62 ± 7.11 *t*_(1215)_ = 0.19, *p* = 0.853	20.97 ± 7.31/20.78 ± 7.15 *t*_(1648)_ = −0.54, *p* *=* 0.589
Openness to experience (NEO-O)	31.42 ± 5.86/32.94 ± 5.78 *t*_(431)_ = 2.68, *p* = 0.008	31.35 ± 6.15/33.02 ± 6.59 *t*_(1215)_ = 4.57, *p* = 5.4 × 10^−06^	31.37 ± 6.07/33.00 ± 6.4 *t*_(1648)_ = 5.3, *p* = 1.3 × 10^−07^
Picture rating task *N*	902	1267	2169
Picture memory task *N*	902	1267	2169

Sample characteristics for the two samples separately and the combined data set

The subjects included in this study represent subsets of two ongoing studies, which were previously described [12, 28]. The purpose of both studies is to identify biological correlates of cognitive performance by using genetics, electroencephalography (EEG), and imaging techniques in healthy young adults from the general population; HC-status was not a primary outcome variable.

### Behavioural tasks descriptions

Subjects in both samples performed on identical versions of two behavioural tasks of interest, namely a picture rating task and a memory task. We only included women with complete data for both tasks and known HC-status (see Table 1). The *picture rating task* consisted of the presentation of *N* = 24 pictures per valence category (negative, neutral, and positive). On the basis of normative valence scores pictures from the International Affective Picture System (IAPS) [29] were assigned to emotionally negative, neutral, and positive picture valence category (negative: 1.4–3.5, neutral: 4.4–5.6, positive: 7.1–8.3). Eight neutral pictures were selected from in-house standardised pictures sets to equate the picture set for visual complexity and content (e.g., human presence). Subjects rated the presented pictures according to valence (negative, neutral, positive) and arousal (low, middle, high) on a three-point scale. In addition to the emotional pictures, 24 scrambled pictures were presented to the subjects in the picture rating task. The background of the scramble pictures contained the colour information of all pictures used in the experiment (except primacy and recency pictures), overlaid with a crystal and distortion filter (Adobe Photoshop CS3). In the foreground, a mostly transparent geometrical object (rectangle or ellipse of different sizes and orientations) was shown. The object had to be rated by the subjects regarding its form (vertical, symmetric, horizontal) and size (small, medium, large).

An unannounced free recall *picture memory task* was the second task of interest. Here subjects had to freely recall the pictures presented during the picture rating task after a 10 min delay. Subjects were instructed to describe the pictures with short keywords, to note as much as they can remember related to the pictures and to describe as many of the pictures as possible. In order to account for primacy and recency effects in memory, two additional pictures showing neutral objects were presented in the beginning and two at the end of the picture rating task. They were not included in the analysis. Two independent and blinded raters scored picture descriptions to identify the number of correctly recalled pictures (Cronbachs alpha 91–98%). A third independent rater then decided for the pictures rated inconsistently.

### Study description

The experiment of sample 1 took place on one visit in combination with magnetic resonance imaging (MRI) data acquisition. Every subject was tested individually. Testing of subjects from sample 2 took place on three visits in groups of 1–7 subjects in a behavioural laboratory in combination with electroencephalography (EEG) measurements. The time interval between visit 1 and 2 was on average 15 days, whereas visit 2 and 3 took place on two consecutive days.

In the following we describe only those parts of the experimental procedure that were relevant for our analyses.

On the first visit, participants received general information about the study and gave their written informed consent. On visit 1 (sample 1) or visit 2 (sample 2) participants were first instructed and then trained on the picture rating task and a working memory task (letter *N*-back [30]). The working memory task served as a distractor between picture rating task and the memory task (see Heck et al. [28] for detailed working memory task description). After training, participants performed on the picture rating task for ~20 min and the distraction task for ~10 min. For sample 1 both tasks were done in the MR-scanner, participants left the MR-scanner after completing the distraction task. Next, participants performed the unannounced free recall picture memory task (no time limit) outside the MR-scanner. Subjects of sample 2 performed all tasks in a behavioural laboratory. The total length of the experimental procedure at visit 1 in sample 1 was ~3–4.5 h and at visit 2 in sample 2 was ~3 h. Participants received 25 CHF/h for participation.

### Statistical analyses

To account for differences across samples we *z*-transformed all task performances (valence and arousal ratings, memory performance) for each sample separately and then data of sample 1 and sample 2 were pooled together.

Ratings (valence and arousal) and memory performance were analysed by calculating three main (mixed) models with subject as random effect, and HC-status (HC-yes/HC-no; between-factor), valence category (negative, positive and neutral; within-factor), and the interaction term between HC-status and valence category as contrasts of interest (fixed effects). The models were estimated by restricted maximum-likelihood estimation (REML). Age was included as covariate in all models to account for the small, but significant group differences in age between the two HC-status groups (Table 1). Statistical tests for significance were done with *F-*tests. In case of significant interaction between HC-status and valence category, post hoc tests for each picture valence category were conducted separately by means of linear models (*t-*test), with HC-status as the variable of interest. For group comparisons (HC-yes vs. HC-no) we estimated Cohen’s *d* as effect size measurement. The estimate of *d* was based on the *t*-value of the linear models, but not on the mean and standard deviation of the respective task performance. Therefore, *d* is corrected for the effects of all confounding variables included in the linear model. By convention, *d* = 0.2 is considered to be a small, *d* = 0.5 to be an intermediate and *d* = 0.8 to be a large effect [31]. Due to the factor coding in our analyses, a positive *d* indicates that the HC-yes group scored higher on a given phenotype compared to the HC-no group. For the mixed models effects, which include a repeated measurement, we report the generalised *η*^2^ [32]. An *η*^2^ = 2% is considered to be small, *η*^2^ = 15% is considered to be intermediate, and *η*^2^ = 35% to be a large effect [31].

Further, we performed some additional analyses. In order to test for differences between samples, we recalculated the three main (mixed) models per sample and for the two samples together using the raw values including the variable sample (sample 1, sample 2). Additionally, to account for differences in personality traits, we first checked for differences between HC-status groups in the values of the five NEO-FFI scales (a Bonferroni corrected *p*-value of <0.01 was considered as significant given the five scales of the NEO-FFI) and then included the scales, which survived the Bonferroni correction (NEO-C and NEO-O) in the three main (mixed) models.

All calculations were done in R [33], the mixed model calculations were done with the nlme package [34], calculations of the generalised *η*^2^ were done with the ezANOVA package [35], and the mediation calculations were done with the MBESS package [36]. All models were calculated with full datasets per subject, which results in an orthogonal design regarding factors with repeated measurements. For the mixed models, all reported *p*-values are nominal *p*-values. For the mediation analysis, the *p*-value of the indirect effect was based on a bootstrapping procedure [36]. Due to the three phenotypes of interest (valence and arousal ratings, memory performance), the significance threshold was set to *p*-value < 0.017 (Bonferroni correction for three independent tests). *p* values <  2.2 × 10^–16^ were not expressed with exact values.

## Results

### Picture rating task

#### Valence rating

**Fig. 1:**
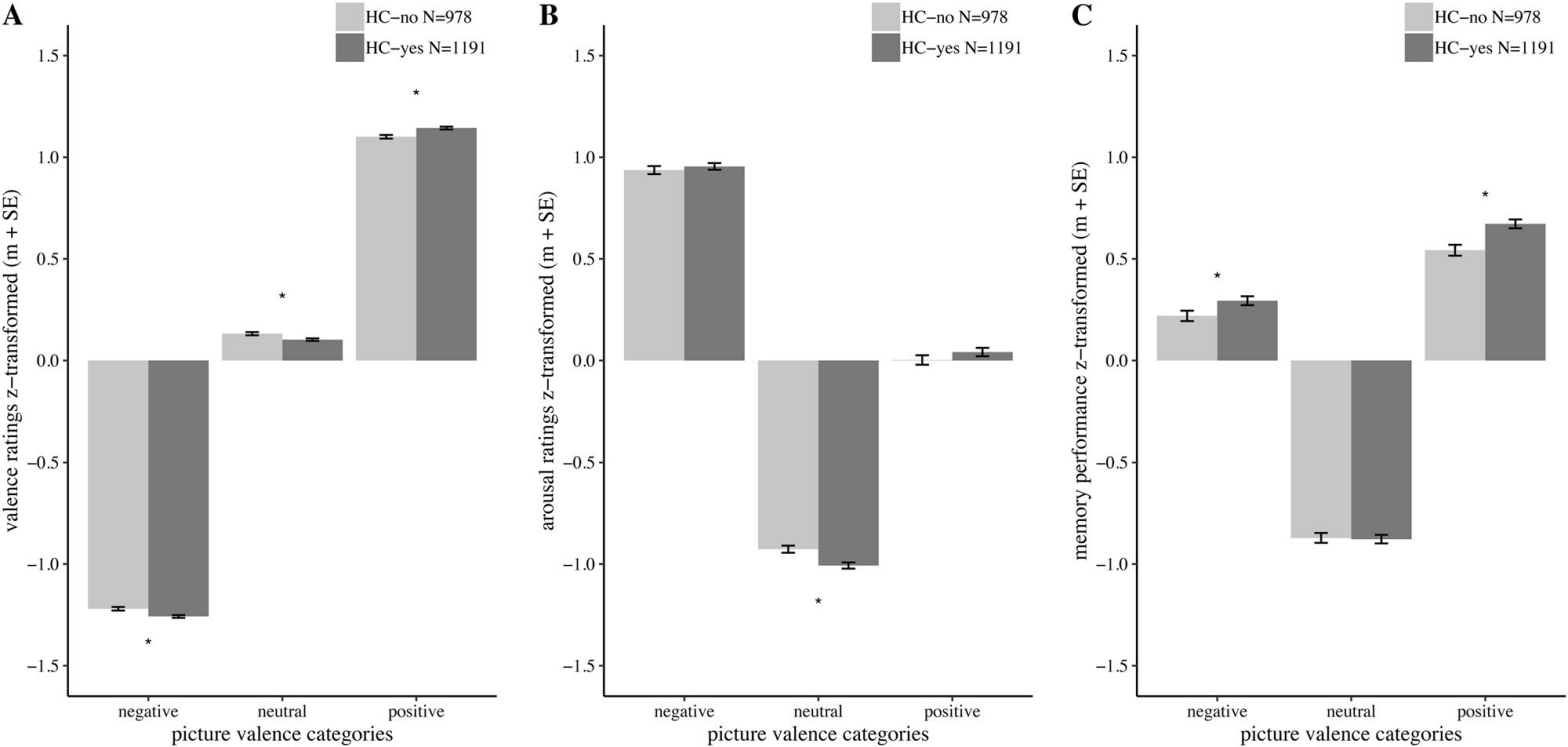
Results of the picture rating task and picture memory tasks. The task performances are z-transformed, therefore a negative task performance denotes that the performance in this group was lower than the average performance. **a** Picture valence rating. **b** Picture arousal rating. **c** memory performance. *m* ± SE = mean and standard error of the mean

Across both HC-status groups, subjects’ averaged valence ratings showed substantial differences between picture valence categories (main effect of valence category: *F*_(2,4334)_ = 49,740.39, *p* < 2.2 × 10^−16^). *Post hoc* tests showed that pictures from the emotional valence categories were significantly more extremely rated compared to neutral pictures (positive vs. neutral: *t*_(4336)_ = −135.91, *p* < 2.2 × 10^−16^; negative vs. neutral: *t*_(4336)_ = −184.63, *p* < 2.2 × 10^−16^). The interaction between HC-status and picture valence category on the valence rating was significant (*F*_(2,4334)_ = 17.54, *p* = 2.59 × 10^−08^, *η*^2^ *=* 0.5%). *Post hoc* tests for each picture valence category separately showed that HC-yes women rated the valence of negative (*t*_(2167)_ = 3.50, *p* = 0.0005, *d* = 0.15), as well as positive pictures (*t*_(2167)_ = −3.90, *p* = 0.0001, *d* = −0.17) as more extreme than HC-no women. Further, HC-yes women rated the valence of neutral stimuli as more neutral than HC-no women (*t*_(2167)_ = 2.94, *p* = 0.003, *d* = 0.13), with the latter rating them towards a positive valence perception. For an overview see Fig. 1a. For the results of the analyses per sample see supplementary materials and methods Table S1. Including the personality scales NEO-C and NEO-O into the model as covariates revealed a similar result (interaction effect: *F*_(2,3296)_ = 16.95, *p* = 4.7 × 10^−08^, *η*^2^ = 0.7%; for details see supplementary materials and methods Table S2).

#### Arousal rating

Across both HC-status groups, subjects’ averaged arousal ratings showed substantial differences between picture valence categories (main effect of valence category: *F*_(2,4334)_ = 10,042.18, *p* < 2.2 × 10^−16^). *Post hoc* tests showed that pictures from the emotional valence categories were significantly more extremely rated compared to neutral pictures (positive vs. neutral: *t*_(4336)_ = −51.47, *p* < 2.2 × 10^−16^; negative vs. neutral: *t*_(4336)_ = 111.73, *p* < 2.2 × 10^−16^). The interaction between HC-status and picture valence category on the arousal rating was significant (*F*_(2,4334)_ = 11.09, *p* = 1.57 × 10^−05^, *η*^2^ = 0.2%). *Post hoc* tests for each picture valence category separately showed no significant differences between the two HC-status groups neither for the arousal rating of negative (*t*_(2167)_ = −0.70, *p* = 0.483, *d* = −0.03) nor positive pictures (*t*_(2167)_ = −1.24, *p* = 0.214, *d* = −0.05). However, the HC-yes group rated neutral pictures as less arousing compared to the HC-no group (*t*_(2167)_ = 3.48, *p* = 0.0005, *d* = 0.15). For an overview see Fig. 1b. For the results of the analyses per sample see supplementary Table S1. The interaction effect (HC-status × valence picture category) was still significant when the NEO-C and NEO-O scales of the NEO-FFI were added as covariates in the model (*F*_(2,3296)_ = 8.54, *p* = 0.0002, *η*^2^ = 0.2%; for details see supplementary materials and methods Table S2).

### Picture memory task

Across both HC-status groups, subjects’ averaged memory performance showed substantial differences between picture valence categories (main effect of valence category: *F*_(2,4334)_ = 3385.47, *p* < 2.2 × 10^−16^). *Post hoc* tests showed that pictures from the emotional valence categories were significantly better remembered than neutral pictures (positive vs. neutral: *t*_(4336)_ = −63.58, *p* < 2.2 × 10^−16^; negative vs. neutral: *t*_(4336)_ = 49.20, *p* < 2.2 × 10^−16^). The interaction between HC-status and picture valence category on the memory performance was significant (*F*_(2,4334)_ = 6.60, *p* = 0.0014, *η*^2^ = 0.1%). *Post hoc* tests for each picture valence category separately revealed that the HC-yes group recalled significantly more positive pictures (*t*_(2167)_ = −3.78, *p* = 0.0002, *d* = −0.16) and nominally significant more negative pictures (*t*_(2167)_ = −2.22, *p* = 0.027, *d* = −0.10) in the memory task compared to the HC-no group. Regarding the memory performance of neutral pictures, the two HC-status groups did not differ significantly (*t*_(2167)_ = 0.19, *p* = 0.848, *d* = 0.01). For an overview see Fig. 1c. For the results of the analyses per sample see supplementary materials and methods Table S1. When including NEO-C and NEO-O scales of the NEO-FFI as covariates in the model, the interaction effect (HC-status × valence picture category) showed a nominal trend only (*F*_(2,3296)_ = 2.62, *p* = 0.073, *η*^2^ = 0.1%). Due to the availability of the NEO-FFI data this result was based on a smaller dataset. By recalculating the model without the NEO-FFI scales within this smaller dataset the effect size of the interaction effect stayed the same (*F*_(2,3296)_ = 2.62, *p* = 0.073, *η*^2^ = 0.1%). Therefore, we conclude that the reduction in significance is due to the substantial reduction in sample size and not due to the inclusion of the NEO-FFI scales into the model (for details see supplementary materials and methods Table S2).

### Mediation analyses

We conducted mediation analyses [37, 38] in order to examine whether the association between HC-status and memory performance was mediated by the observed differences in picture valence ratings. Due to the observed HC-group differences in emotional memory performance (positive and negative), two mediation analyses were performed one for each emotional picture category separately.

**Fig. 2:**
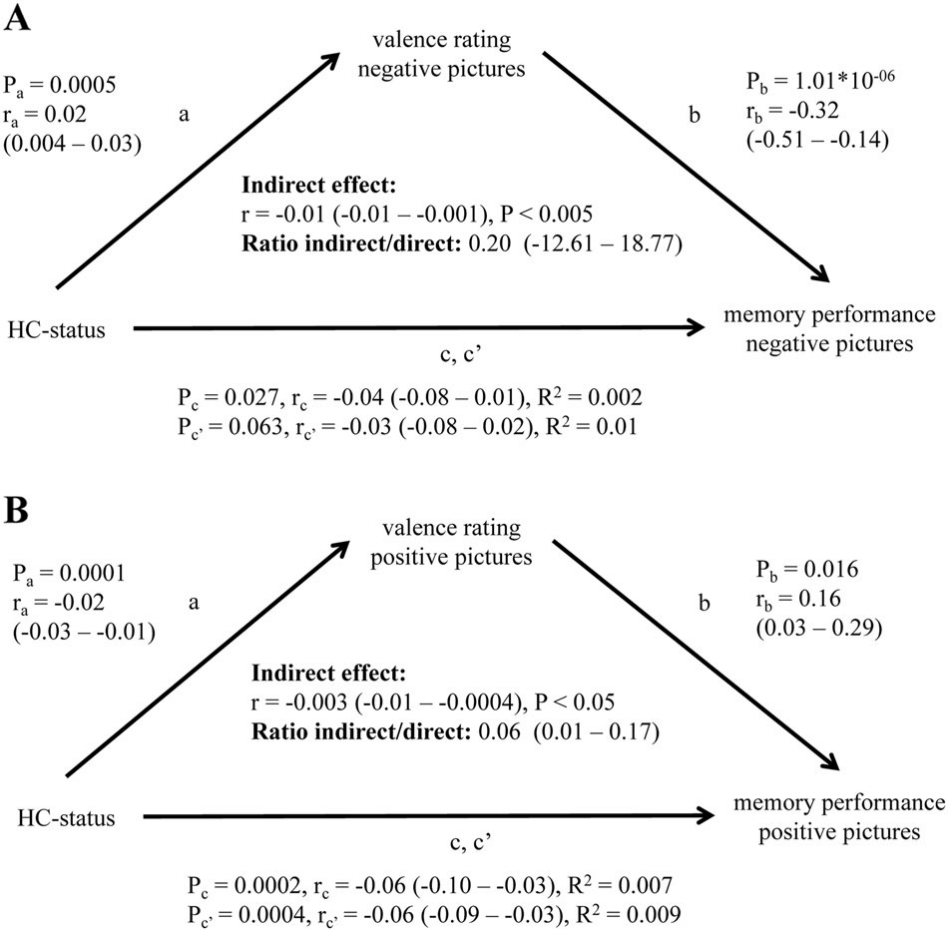
Results of the mediation analyses, testing if the valence rating mediates the association between HC-status and memory performance for the two emotional picture categories separately. (**a**) Negative picture category and (**b**) positive picture category. Path (a) represents the effect of HC-status on valence rating, whereas path (b) is the effect of valence rating on memory performance after removing the effect of HC-status. The indirect effect is computed by multiplying the effects of (a) and (b). Path (c) denotes the effect of the HC-status on memory performance. Path (c’) represents the effect of HC-status on memory performance while controlling for the indirect effect. Parameters (*r*) show the association strength (± 95% confidence interval). Ratio indirect/direct represents the strength of mediation ((a × b)/c’). Parameters (*P*) show significance for path (a), (b), (c), (c’) and indirect effect

There was a significant negative correlation between HC-status and memory performance for both picture valence categories (path c; positive: *r* = −0.06, *p* = 0.0002; negative: *r* = −0.04, *p* = 0.027). For the negative picture category, this association was significantly mediated by the negative picture valence ratings (indirect effect: *r* = −0.01, *p* < 0.005; Fig. 2a). For the positive picture category, the association was partially, albeit nominally significant, mediated by the positive picture valence ratings (indirect effect: *r* = −0.003, *p* *<* 0.05; Fig. 2b).

## Discussion

In this study, we showed that women using HC rated negative pictures as more negative and positive pictures as more positive than women currently not using HC. In addition to these differences in valence ratings of emotional information, HC-users had superior emotional (both negative and positive) episodic memory compared to HC-non-users. Furthermore, the association between HC-status and memory performance was partially mediated by the differences in perceived emotional valence between the HC-status groups. The results from the mediation analyses imply that the more extremely rated valence of emotional pictures in HC-users explains part of the observed better emotional memory performance in this group. To our knowledge this is the first study investigating differences between HC-users and non-users in emotional valence perception, and linking these differences to the observed differences in emotional memory performance. Our results suggest that the well-known memory enhancing effect of emotion, by which emotional information is better remembered than neutral one [10, 11], is more pronounced in women using HC compared to women not using HC.

The herein reported results complement findings from a previous study conducted in our lab about sex-dependent differences in emotional valence and memory [12]. In that study, we had observed a more extreme rating of emotional pictures (positive and negative) in women compared to men (for both, valence and arousal ratings). Women outperformed men in general on the memory task independent of the valence of the material, but with a particular advantage in the recall of positive pictures. In addition to those results, the present study suggests that the intake of HC influences emotional valence perception and thereby explains to certain extent performance differences between women.

Our observation of more extreme emotional valence ratings in HC-users points towards a more intense perception of both negative, as well as positive material. Our results therefore complement the literature reporting mood worsening as well as improvement in OC-users [25, 26]. In the current literature results about HC-effects on emotional processes are inconsistent with some studies reporting either positive [39], negative [15, 40], both [25, 26], or no effects [25]. A possible reason for this heterogeneity may be that emotional processes refer to very diverse sub-processes like perception, processing, recognition, or mood. In future research well-powered, double-blinded, placebo-controlled randomised trials would be desirable, ideally considering different types of HC and using several well-defined phenotypes of emotional processing. Concerning emotional memory, our results of superior memory for both negative and positive pictures in HC-users, suggest that HC-use increases the memory boost for emotional information, which is in line with the results of the existing literature [15, 19, 21, 22].

Our finding of more extreme emotional valence perception and increased emotional memory in HC-users is especially relevant in the context of women’s increased vulnerability to develop mood and anxiety disorders [3–6]. In two recent studies, Skovlund et al. [41, 42] found an association between HC-use and the subsequent use of antidepressants, first diagnosis of depression as well as with subsequent suicide attempt and actual suicide, especially in adolescent women. In accordance with these results, a growing volume of literature emphasises the role of oestrogen and progesterone in anxiety disorders [43–45]. Particular attention is directed towards the effect of oestrogen on extinction memory, as well as the interaction of the hypothalamic-pituitary-adrenal (HPA) axis with the hypothalamic-pituitary-gonadal (HPG) axis within the context of increased vulnerability for these disorders in women [43–45]. Therefore, the observation that HC-users show a stronger perception of emotional material might explain why these females are more vulnerable to develop post-traumatic stress disorder after experiencing a traumatic event when compared to HC non-users.

A few studies suggest an influence of ovarian hormones on post-traumatic stress symptoms [14, 46]. On one hand, variations in ovarian hormone levels during the menstrual cycle are found to be associated with the amount of trauma-related memories. Specifically, spontaneous intrusive recollections and flashback memories are more common, when the traumatic event occurs during the luteal phase [14]. On the other hand, the intake of emergency HC is related to the amount of experienced post-traumatic stress symptoms after a sexual assault. Namely, victims of sexual assault who received a combined emergency HC (ethinyl estradiol and levonorgestrel) directly after the traumatic event report less post-traumatic stress symptoms compared to the victims who either did not take any HC or those who took a progesterone only emergency HC (levonorgestrel) [46]. Emergency HC is used to prevent pregnancy after unprotected intercourse or contraceptive failure. Its mode of action is similar to the one of conventional HC, but compared to those emergency HC consists in the intake of traditional HC at higher doses in one or two intakes [47, 48].

The present study has some limitations. First, the group of HC-users consisted of women using different types of HC (like OC, spiral, patch). Therefore, it is possible that the effects of HC vary between different types of contraceptives. Second, OC-users were taking different types of compounds. In general, a differentiation between OC compounds is based on their hormonal content, namely combined OC (include mostly ethinylestradiol and progestin) vs. progesterone-only OC (include only progestin). Within the combined OC’s further differentiation is made regarding the androgenic potency of the progestin (androgenic vs. anti-androgenic). Some progestins like levonorgestrel bind to androgen receptors (androgenic-combined OC), whereas others such as drospirenone are sensitive specifically to progesterone receptors (anti-androgenic combined OC) [49–52]. Differences in performance on cognitive tasks are observed due to androgenicity of the combined OC’s used [18, 53]. Due to the lack of information about the specific compounds used in our study, we were not able to conduct these sub-type analyses. Furthermore, potential confounding effects due to different reasons for HC-use, prior use of HC (in the HC-no group), HC-intake duration, changes between compounds (in the HC-yes group), and hormone levels cannot be ruled out in our study, due to lack of information on these potentially influencing factors. Additionally, the cross-sectional design of our study could be prone to confounding variables like difference in lifestyle (i.e. relationship status) and personality features between the HC-groups [15]. However, when controlling for differences in personality factors, we did not observe a change in the observed interaction effects for the arousal and valence ratings. For the picture memory task, the interaction between HC-status and picture valence category still showed a nominal trend after the inclusion of the NEO-FFI scales. While it is plausible that the observed interaction effects are related to hormones, it has to be kept in mind that our study describes group differences between women with different HC-status, therefore does not provide direct causal evidence for it.

Our study was well powered given the large sample size (*N* = 2169) [54, 55]. The small effect sizes of the here observed findings are not surprising, as one would not expect a single factor to explain a large proportion of the observed variation of complex cognitive traits [56–58]. Nevertheless, even at small effect sizes, a continuous alteration of information processing under HC-intake may have a large impact on behaviour. Whether this is the case and whether such behavioural changes may be of clinical relevance, we cannot tell from the current data.

In conclusion, our results provide further evidence for altered emotional valence perception and subsequent memory performance in association with HC-intake, based on data from a large sample of 1191 HC-users and 978 HC-non-users. In addition, differences in memory performance between HC-users vs. HC-non-users were partially mediated by differences in emotional valence perception. Therefore, our findings herein underline the importance to consider HC-status in cognitive domains like perception of emotional valence and emotional memory. Moreover, our results provide further evidence about the interrelation of HC-intake with emotional valence perception and emotional memory. Therefore, they might contribute to the understanding of the role of ovarian hormone variations in emotional dysregulation in psychiatric disorders like anxiety and mood disorders, which are more common among women.

## Funding and disclosure

This work was funded by the University of Basel, the Swiss National Science Foundation (grants 163434, 147570 and 159740 to D.J.-F.d.Q. and A.P.), the European Community’s Seventh Framework Programme (FP7/2007–2013) under grant agreement 602450 (IMAGEMEND; grant to A.P. and D.J.-F.d.Q.). The authors declare no competing interests.

## Supplementary information

Table S1

Table S2
